# Linking sexual and reproductive health and HIV interventions: a systematic review

**DOI:** 10.1186/1758-2652-13-26

**Published:** 2010-07-19

**Authors:** Caitlin E Kennedy, Alicen B Spaulding, Deborah Bain Brickley, Lucy Almers, Joy Mirjahangir, Laura Packel, Gail E Kennedy, Michael Mbizvo, Lynn Collins, Kevin Osborne

**Affiliations:** 1 Johns Hopkins Bloomberg School of Public Health, Department of International Health, Baltimore, USA; 2 University of Minnesota School of Public Health, Division of Epidemiology and Community Health, Minneapolis, USA; 3 University of California, San Francisco, Global Health Sciences, San Francisco, USA; 4 World Health Organization, Reproductive Health and Research, Geneva, Switzerland; 5 United Nations Population Fund, New York, USA; 6 International Planned Parenthood Federation, London, UK

## Abstract

**Background:**

The international community agrees that the Millennium Development Goals will not be achieved without ensuring universal access to both sexual and reproductive health (SRH) services and HIV/AIDS prevention, treatment, care and support. Recently, there has been increasing awareness and discussion of the possible benefits of linkages between SRH and HIV programmes at the policy, systems and service delivery levels. However, the evidence for the efficacy of these linkages has not been systematically assessed.

**Methods:**

We conducted a systematic review of the evidence for interventions linking SRH and HIV. Structured methods were employed for searching, screening and data extraction. Studies from 1990 to 2007 reporting pre-post or multi-arm evaluation data from SRH-HIV linkage interventions were included. Study design rigour was scored on a nine-point scale. Unpublished programme reports were gathered as "promising practices".

**Results:**

Of more than 50,000 citations identified, 185 studies were included in the review and 35 were analyzed. These studies had heterogeneous interventions, populations, objectives, study designs, rigour and measured outcomes. SRH-HIV linkage interventions were generally considered beneficial and feasible. The majority of studies showed improvements in all outcomes measured. While there were some mixed results, there were very few negative findings. Generally, positive effects were shown for key outcomes, including HIV incidence, sexually transmitted infection incidence, condom use, contraceptive use, uptake of HIV testing and quality of services. Promising practices (n = 23) tended to evaluate more recent and more comprehensive programmes. Factors promoting effective linkages included stakeholder involvement, capacity building, positive staff attitudes, non-stigmatizing services, and engagement of key populations.

**Conclusions:**

Existing evidence provides support for linkages, although significant gaps in the literature remain. Policy makers, programme managers and researchers should continue to advocate for, support, implement and rigorously evaluate SRH and HIV linkages at the policy, systems and service levels.

## Background

The international community agrees that the Millennium Development Goals will not be achieved without ensuring universal access to both sexual and reproductive health (SRH) services and HIV prevention, treatment, care and support [1]. Recently, there has been increasing awareness and discussion of the possible benefits of linkages between SRH and HIV programmes at the policy, systems and service delivery levels [2-5].

Linkages between SRH and HIV-related policies and programmes may lead to a number of important public health, societal and health systems benefits [2]. Linkages are expected to improve coverage, access to and uptake of both SRH and HIV services for vulnerable and key populations (where HIV risk and vulnerability converge), including people living with HIV (PLHIV) [2]. Linking SRH and HIV interventions may lead to a reduction in HIV-related stigma and discrimination [2] by integrating HIV with other SRH services. Linkages may enhance programme effectiveness and efficiency [2] as redundancies in vertical programmes are eliminated and clients' multiple needs are addressed in one setting [3].

These potential efficiencies and cost savings are particularly important in the context of a maturing global response to HIV that focuses less on emergency measures and more on ensuring long-term sustainability and integration of HIV programmes with other programmes and health systems. Linkages may improve access to family planning and other key SRH services for PLHIV, thereby reducing perinatal transmission with a cost-effective component of prevention of mother to child transmission (PMTCT) [6,7] and ensuring access by PLHIV to SRH services tailored to their needs [8].

The international community has issued statements calling for commitment and action to increase linkages as a result of these and other expected benefits [4,5]. However, prior to this study, the evidence that linkages actually result in these benefits had not been systematically examined. Evidence for the benefits of SRH and HIV linkages is crucial to sound funding, programmatic and policy decisions.

There have been several compilations of articles and reports related to SRH and HIV linkages. These include an inventory of documents and tools related to SRH-HIV linkages [9] and a continuously updated website compiling full-text documents, tools, news reports and other resources [10]. Despite these resources, evidence in support of linkages has not been rigorously evaluated. This study presents the first systematic review and analysis of interventions linking SRH and HIV.

## Methods

A supplementary file with a more detailed description of methods, including the list of search terms, is available online [11].

### Definitions

Linkages can occur at multiple levels. In order to capture all of these levels, the following definition of linkages was used: "the bi-directional synergies in policy, programmes, services and advocacy between SRH and HIV" [12]. To be included in the review, studies had to meet this definition by evaluating a linkage between an SRH intervention and an HIV intervention. HIV interventions were classified into five categories: (1) HIV prevention, education, and condoms; (2) HIV testing; (3) element 3 of PMTCT (prevention of vertical HIV transmission from a mother to her infant) [13]; (4) clinical care for PLHIV; and (5) psychosocial and other services for PLHIV. Interventions related to injection drug use would generally fall under categories 1 or 5.

SRH interventions were also classified into five categories: (1) family planning; (2) maternal and child health care; (3) gender-based violence prevention and management; (4) sexually transmitted infection (STI) prevention and management; and (5) management of other SRH issues, such as gynaecologic cancers, obstetric fistula and menopause. Studies reporting interventions on element 3 of PMTCT not linked to other areas of SRH were excluded as these interventions have been reviewed elsewhere [14-16].

### Inclusion criteria

An article was included in the review if it met the following criteria:

1. Published in a peer-reviewed journal between 1 January 1990 and 31 December 2007

2. Presents post-intervention evaluation data of an SRH-HIV linkage intervention

3. Used a pre-post or multi-arm comparison of individuals who received the intervention versus those who did not to assess quantitative outcomes of interest (biological, behavioural, knowledge or process outcomes).

Any article meeting these criteria was included in the review, even if the specific research objective was not originally related to linkages. No language restrictions were imposed. Authors were contacted for additional clarification when needed.

In addition, due to the relatively new and dynamic nature of SRH-HIV linkages, we also gathered unpublished programme reports. These were termed "promising practices." Promising practices were included if they had any evaluation data from an SRH-HIV linkage intervention and were limited to studies conducted in low- and middle-income countries, as defined by the World Bank [17]. Including promising practices from low- and middle-income countries only was a limitation of the review. However, given the potentially vast amount of unpublished literature from high-income countries, we felt it was necessary to narrow the scope of the search for promising practices, and chose to focus on the parts of the world for which linkages are most discussed.

### Search strategy

A list of search terms was generated by combining terms related to SRH, HIV and study design. This list was entered into three electronic databases: PubMed (including MEDLINE and AIDSLINE), the Cumulative Index to Nursing and Allied Health Literature (CINAHL), and EMBASE (Excerpta Medica). In addition, the table of contents of 14 journals in the fields of SRH and HIV were hand searched, reference lists of included articles and other key documents were examined, relevant SRH and HIV websites were searched, and experts were contacted to identify additional citations.

### Screening process

Citations were downloaded into bibliographic management software (EndNote V.10) and screened using a three-step process. First, titles and abstracts of all citations were read to exclude those that clearly did not meet the inclusion criteria. Second, remaining citations were double screened by two independent staff members. These screening results were compared and discrepancies resolved through discussion. Third, the full text of included articles was read to ensure correct study classification.

### Data extraction

Each article was read and data were extracted by two members of the study team working independently. Differences in data extraction or interpretation of studies were resolved by discussion and consensus. Data were extracted into tables that recorded the following information: type of linkage, location, setting, target group, years of programme and evaluation, intervention description, study design, unit of analysis, sample size, age and gender of participants, length of follow up, reported numerical outcomes and results, text summary of outcomes, integration direction, study objective, integration format (on site, referral, etc.), promoting factors, inhibiting factors, and author recommendations.

### Outcomes extraction

Following data extraction, study outcomes were classified according to pre-defined outcomes categories. Outcomes extractions were conducted by two individuals independently with resolution by discussion. Results from nine key outcomes are presented. Eight of these were selected *a priori *(HIV incidence, STI incidence, condom use, contraceptive use, uptake of HIV testing, quality of services, stigma and cost), while the ninth (unintended pregnancy) was added based on feedback from presentations of preliminary results.

Each reported outcome was assessed to determine whether that outcome was related to the intervention (i.e., whether the intervention was intended to affect that outcome). Studies where the outcome was considered related to the intervention were then classified based on intervention objectives into studies that had a positive effect, a negative effect, no change, or a mixed effect (used when the study presented either multiple measures of the same outcome or multiple measures over time, and these different measures showed different results).

### Study rigour

Study rigour was assessed using a nine-point scale, with a minimum score (low rigour) of 1 and a maximum score (high rigour) of 9. This scale was adapted from an eight-point rigour assessment scale developed for systematic reviews of HIV behavioural interventions [18]. Studies received one point for meeting each of the following criteria: (1) study design includes pre/post intervention data; (2) study design includes control or comparison group; (3) study design includes cohort; (4) comparison groups equivalent at baseline on socio-demographics; (5) comparison groups equivalent at baseline on outcome measures; (6) random assignment (group or individual) to the intervention; (7) participants randomly selected for assessment; (8) control for potential confounders; and (9) follow-up rate ≥ 75%.

## Results

**Figure 1:**
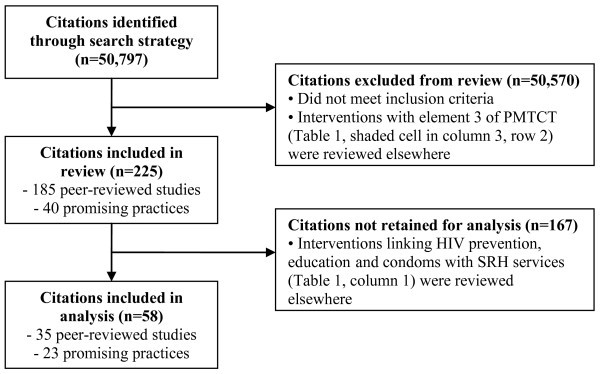
**Flow chart showing disposition of citations**.

Our search strategy identified 50,797 individual citations (Figure 1). Of these, 185 peer-reviewed studies met the inclusion criteria and were included in the review. Table 1 displays the different types of intervention linkages for included articles. Of the 185 included articles, 150 reported on interventions linking SRH with HIV prevention, education and condoms (Table 1, column 1) that were not also included in other categories (Table 1, columns 2-5). These studies were excluded from the analysis as they have been reviewed elsewhere [19-21]. The remaining 35 studies (Table 1, columns 2-5) were included in the analysis [22-56].

**Table 1:** Matrix of results by type of linkage

	HIV prevention, education, & condoms	HIV counselling & testing	Element 3 of PMTCT	Clinical care for PLHIV	Psychosocial & other services for PLHIV
**Family planning**	54	6	2	1	6

**Maternal & child health care**	7	15	N/A	2	1

**GBV prevention & management**	4	1	1	1	0

**STI prevention & management**	129	9	1	4	5

**Other SRH services**	0	1	0	2	1

Note: Several studies included multiple linkages, so the numbers reported in the table exceed the total number of studies included in the review.

GBV: gender-based violence; STI: sexually transmitted infection; SRH: sexual and reproductive health; N/A: not applicable

### Location and populations

The 35 studies included in the analysis covered a wide range of countries and target populations (Additional file 1, Table S1, available online [11]). The region most represented was Africa, with 18 studies located in eight different countries. The remaining studies were located in the United States of America (n = 7), the United Kingdom (n = 4), India (n = 2), Thailand (n = 2), China (n = 1) and Haiti (n = 1). Target populations also varied, and included adult men and women, pregnant women, adolescents, commercial sex workers, people living with HIV and HIV-discordant couples.

### Interventions

Types of interventions varied tremendously, as reflected in the wide distribution of studies across linkage types (Table 1). Most interventions incorporated some form of HIV testing, while fewer included interventions from element 3 of PMTCT, clinical care for PLHIV, or psychosocial and other services for PLHIV; no injection drug use-related interventions were identified. Few interventions were linked with gender-based violence prevention and management or management of other SRH services.

The majority of studies (25 out of 35) reported on interventions that contained only one type of linkage (i.e., fell into only one cell in Table 1). Only three studies covered more than two types of linkages. Of the 35 studies included in the analysis, 18 integrated HIV services into existing SRH services, 12 integrated SRH services into existing HIV services, and five integrated HIV and SRH services concurrently.

### Study rigour

On our nine-point scale, the average rigour score was 3.46 (Table 2). Only six studies used a randomized, controlled design (randomizing either individuals or groups to the intervention). No studies directly compared linked services to the same services offered separately; more often, they compared outcomes before and after a linked service was added to existing services, or they compared an intervention group offering linked services with a comparison group offering services in only one area.

**Table 2:** Study rigour

Study	Study design includes pre-post intervention data	Study design includes control or comparison group	Study design includes cohort	Comparison groups equivalent at baseline on socio-demographics	Comparison groups equivalent at baseline on outcome measures	Random assignment (group or individual) to the intervention	Participants randomly selected for assessment	Control for potential confounders	Follow-up rate ≥ 75%	Total rigour score (min score = 1; max score = 9)
Allen, Serufilira, 1992 [22]	Yes	No	Yes	N/A	N/A	No	Yes	N/A	Yes	4

Allen, 1993 [23]	Yes	No	Yes	N/A	N/A	No	Yes	No	Yes	4

Allen, Tice, 1992 [24]	Yes	No	Yes	N/A	N/A	No	No	N/A	Yes	3

Anderson, 2004 [25]	Yes	No	No	No	N/A	No	No	Yes	N/A	2

Bentley, 1998 [26]	Yes	No	Yes	N/A	N/A	No	No	N/A	Yes	3

Bhave, 1995 [27]	Yes	Yes	Yes	Yes	Yes	No	No	No	Yes	6

Cartoux, 1999 [28]	No	Yes	No	N/A	N/A	No	No	No	N/A	1

Chamot, 1999 [29]	Yes	Yes	Yes	NR	N/A	No	No	Yes	Yes	5

Chandisarewa, 2007 [30]	Yes	Yes	No	N/A	N/A	No	No	No	N/A	2

Clark, 1998 [31]	Yes	No	Yes	N/A	N/A	No	Yes	No	Yes	4

Creanga, 2007 [32]	No	Yes	No	N/A	N/A	No	No	Yes	N/A	2

Coyne, 2007 [33]	Yes	Yes	No	N/A	N/A	No	No	No	N/A	2

Farquhar, 2004 [34]	Yes	Yes	Yes	No	Yes	No	No	Yes	No	5

Ghys, 2002 [35]	Yes	No	No	N/A	N/A	No	No	Yes	N/A	2

Hamlyn, 2007 [36]	Yes	Yes	No	No	N/A	No	No	No	N/A	2

Jones, 2004 [37]	Yes	Yes	Yes	Yes	Yes	Yes	No	No	Yes	7

Jones, 2006 [38]	Yes	Yes	Yes	Yes	Yes	Yes	No	Yes	Yes	8

Khoshnood, 2006 [39]	Yes	Yes	Yes	No	NR	No	No	Yes	Yes	5

Kiarie, 2006 [40]	Yes	No	Yes	N/A	N/A	No	No	No	Yes	3

King, 1995 [41]	Yes	No	Yes	N/A	N/A	No	No	N/A	Yes	3

Kissinger, 1995 [42]	Yes	Yes	Yes	No	Yes	No	No	Yes	Yes	6

McCarthy, 1992 [43]	No	Yes	No	N/A	N/A	No	No	No	N/A	1

Peck, 2003 [44]	Yes	No	No	N/A	N/A	No	No	No	N/A	1

Rasch, 2006 [45]	No	Yes	No	NR	N/A	No	No	Yes	N/A	2

Richardson, 2004 [46]	Yes	Yes	Yes	No	No	Yes	No	Yes	No	5

Semrau, 2005 [47]	No	Yes	No	N/A	N/A	No	No	No	N/A	1

Sherr, 2007 [48]	Yes	Yes	Yes	NR	NR	No	No	Yes	No	4

Simpson, 1998 [49]	No	Yes	No	Yes	N/A	Yes	No	No	N/A	3

Sirivongrangson, 2006 [50]	No	Yes	No	N/A	N/A	No	No	N/A	N/A	1

Stringer, 2007 [51]	Yes	Yes	Yes	Yes	NR	Yes	No	No	No	5

Stringer, 2001 [52]	Yes	Yes	No	No	N/A	No	No	Yes	N/A	3

Stringer, 2003 [53]	No	Yes	No	Yes	N/A	Yes	No	No	Yes	4

Van't Hoog, 2005 [54]	Yes	No	No	N/A	N/A	No	No	No	N/A	1

Wingood, 2004 [55]	Yes	Yes	Yes	Yes	Yes	Yes	No	Yes	Yes	8

Xu, 2002 [56]	Yes	No	Yes	N/A	N/A	No	No	N/A	Yes	3

N/A: not applicable; NR: not reported

### Outcomes

Overall, the majority of studies showed improvements in all outcomes measured (beyond the nine key outcomes). While there were a few mixed results, there were very few negative findings. Twenty-three studies reported at least one of the nine key outcomes.

### HIV incidence

Two studies reported HIV incidence [22,48]. The average rigour score of the two studies was 4. Sherr and colleagues provided free HIV voluntary counselling and testing (VCT) and treatment for other STIs through a mobile clinic [48]. After three years, HIV incidence in the intervention group (tested) was 22.5 per 1000 person years (95% confidence interval 14.2, 36.7), lower than in the first control group (tested but not received results), 23.1 per 1000 person years (95% CI 15.2, 35.0), but higher than in the second control group (never tested), 17.5 per 1000 person years (95% CI 14.8, 20.6). This was categorized as a mixed effect.

Allen and colleagues provided VCT to women recruited from prenatal and paediatric clinics, along with an AIDS educational video, group discussion, and free condoms and spermicide [22]. Results showed a positive effect (e.g., lowered rate of HIV seroconversion) among participants after the intervention (3.0 per 100 person years, 95% CI 2.2, 3.7) compared with before (4.1 per 100 person years, 95% CI 3.0, 5.1).

### STI incidence

Two studies, with an average rigour score of 6.5, reported STI incidence; both showed a positive effect [29,55]. Wingood and colleagues conducted a randomized, controlled trial of an intervention consisting of four weekly interactive group sessions emphasizing female empowerment, supportive networks, HIV risk behaviours, communication, and condom use skills and healthy relationships among HIV-infected women in the United States [55]. At the 12-month follow up, the adjusted odds ratio of incident gonorrhea and Chlamydia comparing intervention with control group participants was 0.1 (95% CI 0.01, 0.6).

Chamot and colleagues offered HIV testing targeting adolescents at a public STI clinic in the United States [29]. Among 22 patients who tested HIV positive after baseline, the rate of gonorrhea dropped by nearly 75% after testing (44.5 per 100 person years before, 12.5 per 100 person years after). Among HIV-negative individuals, the gonorrhea reinfection rate increased with the number of HIV tests experienced during follow up, but follow-up rates were consistently lower than rates prior to the first HIV test.

### Condom use

Ten studies reported condom use as an expected outcome of the intervention (average rigour score = 4.4). Seven studies showed a positive effect on condom use [24,26,27,34,35,55,56]. These studies covered a variety of interventions, including: VCT for male STI clinic attendees [26]; VCT for women attending antenatal or paediatric clinics and their partners [24,34,56]; a behavioural intervention for HIV-infected women [55]; and two clinics that provided a range of SRH and HIV services to commercial sex workers [27,35].

Two studies showed a mixed effect on condom use [33,38]. In one case, after an HIV clinic added family planning services, the use of condoms only as contraception declined from 30% to 7% (significance not reported). However, study authors interpreted this positively as improved provision of more reliable contraceptives [33], so we classified it as a mixed effect. In the second study showing a mixed effect, Jones and colleagues found inconsistent condom use across different follow-up periods after a behavioural intervention with HIV-infected Zambian women [38]. Finally, one study by Sherr and colleagues showed no effect, as there was no change in condom use following free mobile VCT and STI treatment [48].

### Contraceptive use

Four studies reported contraceptive use (other than condoms) as an expected outcome of the intervention (average rigour score = 4.25). One showed a positive effect [41] and three showed a mixed effect [23,38,45]. Two of these studies, one positive and one mixed, were conducted by the same research group. While both provided family planning information to women receiving VCT in Rwanda, one showed a significant improvement in hormonal contraceptive use (16% to 24%, p = 0.002) [41], while the other showed mixed effects, as hormonal contraceptive use decreased among HIV-infected women (23% to 16%), but not among HIV-negative women (17% to 18%) (significance not reported) [23]. In the other two studies, contraceptive use was measured against or in combination with condom use, making it difficult to interpret outcomes for contraceptive use alone [38,45].

### Uptake of HIV testing

Nine studies reported uptake of HIV testing as an outcome related to the intervention (average rigour score = 2.22); all showed a positive intervention effect on uptake of HIV testing [25,30,43,44,48,49,52,54,56].

### Quality of services

Four studies reported some measure of quality of services as an outcome related to the intervention (average rigour score = 3.0). Three studies measuring provider implementation of consultation procedures showed a positive effect [33,36,46], while one study measuring client satisfaction showed no effect [49].

### Unintended pregnancy, stigma, and cost

No studies measured unintended pregnancy, stigma or cost as expected outcomes of the intervention.

### Promising practices

Twenty-three promising practices were analyzed as part of the review [11]. These articles and reports from the grey literature generally evaluated more recent and more comprehensive interventions than the peer-reviewed studies. For example, while most peer-reviewed studies covered only one type of linkage, promising practices frequently covered five, six, seven or more linkage categories. Although promising practices generally employed less rigorous study designs, the intervention objectives often more closely matched the goals described by individuals and organizations working to promote SRH/HIV linkages where appropriate.

Overall, findings from promising practices were similar to findings from peer-reviewed studies. Some promising practices reported cost, and suggested potential cost savings from linkages. However, cost-reporting data and cost-effectiveness methodologies were generally weak. Quality of service measures were more varied than in peer-reviewed articles, and included quality checklists and multiple quality outcomes.

### Promoting and inhibiting factors

Factors promoting and inhibiting successful integration, as reported by study authors, were examined for both peer-reviewed studies and promising practices. Promoting factors included: stakeholder involvement; capacity building; positive staff attitudes and non-stigmatizing services; and engagement of key populations. Inhibiting factors included: lack of sustainable funding and stakeholder commitment; staff shortages, high turnover, and inadequate staff training; poor programme management and supervision; inadequate infrastructure, equipment, and commodity supply; and client barriers to service utilization, including low literacy, lack of male partner involvement, stigma, and lack of women's empowerment to make SRH decisions.

## Discussion

Overall, the majority of studies showed improvements in all outcomes measured. Linking SRH and HIV services was considered beneficial and feasible. Linkages showed generally positive effects on HIV incidence, STI incidence, condom use, uptake of HIV testing and quality of services. There were some mixed effects, particularly with contraceptive use, but this was largely due to contraceptive use measures that were compared with or combined with condom use measures, making findings difficult to interpret. This highlights the importance of considering both HIV- and SRH-related goals when selecting outcomes for assessment, specifically dual-method use. Overall, there were very few negative outcomes. No studies measured unintended pregnancy, stigma or cost.

Although this review included a large number of studies, it also identified several gaps in the existing evidence. Inadequately studied interventions included linked services targeting men and boys, services addressing gender-based violence prevention and management, and comprehensive SRH services for PLHIV. Insufficiently reported outcomes included health, stigma and cost outcomes. Infrequently used study designs and research questions included research questions that specifically address SRH and HIV service integration and study designs that compare integrated services with the same services offered separately.

This is an important point: while studies included in this review technically met our inclusion criteria and definition of linkages, they often focused on research questions that were not the most important questions for individuals specifically concerned with linkages. In addition, while we would have included linkages at any level (policy, systems or service delivery), nearly all interventions included were at the service delivery level. Linkages at the policy and systems levels are unlikely to be evaluated using the same rigorous designs as service delivery linkages.

In an attempt to identify all potentially relevant articles and reports, our search included unpublished programme reports. Conclusions based on these promising practices are tentative due to generally weak study designs and the difficulty of identifying unpublished reports. Despite these limitations, promising practices often evaluated programmes with objectives that more closely match the broader field of SRH-HIV linkages and thus provided more useful lessons learned. Promising practices also tended to evaluate more recent and more comprehensive programmes (i.e., interventions covering more types of linkages) than peer-reviewed studies. This may indicate that more recent programmes linking SRH and HIV are more comprehensive in scope.

The strengths of this review include its systematic methodology and broad scope, covering the entire field of SRH and HIV linkages. However, because this review was so broad in scope, the included studies varied enormously in terms of types of interventions, target populations, research questions and objectives, study designs, rigour and outcomes. Such heterogeneity made it difficult to synthesize data across studies, and difficult to make concrete recommendations about which types of linkages work best and in which settings. Not all linkages will make sense in all settings, and programme planners must carefully consider multiple factors, including target population, local HIV and SRH context, and programme resources, goals, opportunities and challenges when deciding how to operationalize linkages. In addition, although we made an attempt to search and include unpublished reports as promising practices, our search strategy most likely did not capture all documents that would have met the inclusion criteria, specifically older reports that are not permanently archived.

To facilitate use of findings by programme planners, we have created an eight-page summary document that presents findings from this review by type of programme to facilitate comparisons with existing programmes; this document is available on the WHO, UNFPA and UNAIDS websites [57]. In addition, the subset of studies evaluating family planning and HIV linkages has been examined in greater detail separately [58].

## Conclusions

Despite its limitations, the strengths of this review allow several recommendations to be made to policy makers, programme managers and researchers. Policy makers should advocate for and support SRH and HIV linkages at the policy, systems and service levels, since they are demonstrated to improve outcomes. Programme managers should strengthen linked SRH and HIV responses in both directions where feasible and appropriate, and then rigorously monitor and evaluate integrated programmes during all phases of implementation. Researchers should direct rigorous research efforts toward linkages that are currently understudied, evaluate key outcomes and disseminate findings.

## Competing interests

The authors declare that they have no competing interests.

## Authors' contributions

CK served as lead study coordinator and coordinator for peer-reviewed studies, co-led design of the study protocol, conducted online database searches, screened and extracted data from peer-reviewed articles, and drafted the manuscript. AS critically reviewed the study protocol, and screened and extracted data from peer-reviewed articles. DBB screened and extracted data from promising practices. LA served as coordinator for promising practices, and screened and extracted data from promising practices. JM screened and extracted data from promising practices. LP co-led design of the study protocol, and screened promising practices. GK served as overall project coordinator, assisted with design of the study protocol, and screened and extracted data from promising practices. MM, LC and KO conceptualized the study, and critically reviewed the study protocol. All authors assisted with analysis and interpretation of the data, reviewed the manuscript for important intellectual content, and provided final approval of the version submitted for publication.

## Authors' information

CK is an Assistant Professor in the Department of International Health, Social and Behavioral Interventions Program at the Johns Hopkins Bloomberg School of Public Health. AS is a doctoral student at the University of Minnesota School of Public Health, Division of Epidemiology and Community Health DBB, JM, LP and GK are with the Cochrane Collaborative Review Group on HIV Infection and AIDS (Cochrane HIV/AIDS Group) at the Prevention and Public Health Group, Global Health Sciences at the University of California, San Francisco. LA was with the Cochrane HIV/AIDS Group and is now an MPH student at Columbia University, Mailman School of Public Health. MM is with the World Health Organization, Division of Reproductive Health and Research. LC is with the United Nations Population Fund. KO is with the International Planned Parenthood Federation.

## Supplementary Material

Additional file 1 **Table S1**. Study description table. Click here for file
