# Supplementary material for: Linking sexual and reproductive health and HIV interventions: a systematic review
Source: J Int AIDS Soc. 2010 Jul 19;13:26. doi: 10.1186/1758-2652-13-26 (PMC2918569; doi:10.1186/1758-2652-13-26)
Supplement: Additional file 1 — Table S1. Study description table. [file 1758-2652-13-26-S1.DOC]

**Table S1. Study description table**

| **Study** | **Country, target population, and setting** | **Types* and direction of linkages** | **Intervention description** | **Study design and rigour score** | **Key outcomes reported** |
| --- | --- | --- | --- | --- | --- |
| Allen, Serufilira, 1992 [22] | Rwanda  Women attending prenatal and paediatric clinics  Study clinic | L7, L17  SRH services adding HIV services | Voluntary counselling and testing for HIV was provided to women recruited from prenatal and paediatric clinics. One week after enrolment, women watched a 35-minute AIDS educational video in groups of 10 to 15, followed by a group discussion led by a physician and a social worker. Condoms and spermicide were distributed at no charge. At the request of study subjects. male partners were free to request the test but were not required to have it. | Time series study design  Rigour score = 4 | HIV incidence |
| Allen, 1993 [23] | Rwanda  Pregnant/childbearing women  ANC and paediatric clinics | L1, L2, L6  Simultaneous | HIV testing was provided to women attending ANC or paediatric clinics. Women watched a 35-minute AIDS educational video, followed by a group discussion. Condoms and spermicide were distributed at no charge. Male partners were free to request HIV testing and attend the video and group discussions. Risk-reduction counselling was provided for HIV-negative women and in-depth counselling for HIV-positive women, including some discussion of family planning measures, though this was not pursued unless indicated by the women. | Time series study design  Rigour score = 4 | Contraceptive use |
| Allen, Tice, 1992 [24] | Rwanda  Discordant couples recruited from women attending prenatal and paediatric clinics  Study clinic | L7  SRH services adding HIV services | Couples watched an AIDS education video and attended a group discussion led by a social worker. Condoms and spermicide were provided free of charge. VCT was conducted and HIV results were given individually in sealed envelopes. Couples were encouraged to receive their results together. | Pre-post study design  Rigour score = 3 | Condom use |
| Anderson, 2004 [25] | USA  Pregnant women  Prenatal clinic | L7  SRH services adding HIV services | An ANC clinic added an HIV-focused educational nurse to their staff. The nurse presented a brief, standardized educational programme to staff, focusing on the importance of universal screening for HIV and non-confrontational methods of patient education and counselling, with the goal of improving testing acceptance rates in the clinic. This was followed by the continued presence of the nurse in the clinics and her availability to coordinate medical and social care for HIV-positive clients and their infants. | Serial cross-sectional study design  Rigour score = 2 | Uptake of HIV testing |
| Bentley, 1998 [26] | India  Men  STI clinics | L17  SRH services adding HIV services | VCT was provided for HIV-negative men recruited from STI clinics. Individual pre-test counselling was provided by clinic social workers and covered such topics as HIV transmission routes, risk behaviours, prevention strategies and a condom demonstration. Condoms were provided free of cost. During subsequent visits, men were examined for STIs and counselled on abstinence or condom use until their current STI was cured and their HIV test was confirmed as negative. | Time series study design  Rigour score = 3 | Condom use |
| Bhave, 1995 [27] | India  Female commercial sex workers (CSWs) and madams in brothels  Study clinics for CSWs | L17  Simultaneous | A clinic providing services only for CSWs was established in a red-light area. HIV testing and counselling and testing for syphilis and hepatitis B were conducted for all women. Women complaining of symptoms underwent a pelvic examination by a gynecologist, and were evaluated, treated or referred for STIs as necessary. The intervention consisted of educational videos, small group discussions, and use of pictoral educational materials. A separate educational intervention was conducted with brothel madams. Free condoms were provided (50/person/week). | Non-randomized trial – group  Rigour score = 6 | Condom use |
| Cartoux, 1999 [28] | Burkina Faso  Pregnant women  Antenatal clinics | L7  SRH services adding HIV services | Women attending antenatal clinics were provided VCT in either group or individual counselling sessions. HIV knowledge was reinforced at pre-test sessions and evaluated at post-test sessions. Pre-test counselling was performed in the local language by social workers in a group during the first six months of the study and individually during the following year. | Cross-sectional study design  Rigour score = 1 | None |
| Chamot, 1999 [29] | USA  Adolescents and young adults  STI clinic | L17  SRH services adding HIV services | At the largest public outpatient STI clinic in New Orleans, all patients were offered HIV tests. For much of the study period, pre-test counselling was short (<5 min), and post-test counselling was rarely perfomed. Patients were not encouraged to come back to the clinic for test results, but were told that “no news from the clinic means good news”. The aim of the study was to examine the effect of HIV testing on gonorrhea incidence. | Retrospective cohort  Rigour score = 5 | STI incidence |
| Chandisarewa, 2007 [30] | Zimbabwe  Pregnant women  Antenatal clinics | L7  SRH services adding HIV services | Provider-initiated, opt-out HIV testing with right of refusal was offered to all new ANC clients, starting in June 2005. Before this, HIV testing was opt-in. Community mobilization activities were conducted, including a skit performed in numerous settings. Group education/discussion was conducted prior to testing. Extensive individual post-test counselling was conducted. Any woman refusing testing was extensively counselled. Infant feeding counselling was conducted as appropriate to HIV status. | Serial cross-sectional study design  Rigour score = 2 | Uptake of HIV testing |
| Clark, 1998 [31] | USA  Adolescents  Adolescent medicine clinic | L17  SRH services adding HIV services | At an adolescent medicine clinic, patients were offered HIV testing and counselling and reinforcement of safer sex practices. | Pre-post study design  Rigour score = 4 | None |
| Creanga, 2007 [32] | Ethiopia  Community-based reproductive health agents  Community-based | L1, L2, L5  SRH services adding HIV services | Community-based reproductive health agents provided family planning education and methods (including condom distribution), HIV education, referral to VCT, and home-based care for PLHIV. They provided health outreach services to households, often in rural areas, on a voluntary basis, though it was common for them to receive non-monetary incentives, such as uniforms, supplies, and travel reimbursement. | Cross-sectional study design  Rigour score = 2 | None |
| Coyne, 2007 [33] | United Kingdom  HIV-positive women  HIV clinic | L5, L10, L20, L25  HIV services adding SRH services | The Garden Clinic, for HIV-positive women, started a specific clinic (FP Plus) to provide HIV-positive women clients with screening for STIs, contraception, pre-conception counselling, and cervical cytology. The Garden Clinic already worked on a model of integrated sexual health care, and FP Plus is staffed by doctors and senior nurses trained in both STI management and family planning. | Serial cross-sectional study design  Rigour score = 2 | Condom use  Quality of services |
| Farquhar, 2004 [34] | Kenya  Pregnant women and their partners  Antenatal clinic | L7  SRH services adding HIV services | Women attending an antenatal clinic were invited to participate, baseline data was collected, and the pros and cons of partner testing, couple VCT and partner notification were discussed individually. All women were invited back to the clinic one week later for counselling and testing. Women who chose to return to the clinic with their partners were offered either couple counselling or counselling as individuals. Women who returned alone were offered individual counselling and testing. Both men and women were asked to return in two weeks for additional counselling relevant to their HIV status (e.g., breastfeeding, nevirapine). | Non-randomized trial – individual  Rigour score = 5 | Condom use |
| Ghys, 2002 [35] | Côte d’Ivoire  Female commercial sex workers (CSWs)  Study clinic for HIV/STIs | L17  Simultaneous | A prevention campaign targeted at female CSWs included peer education and group health education sessions conducted by current and former CSWs in bars, hotels and other sex-work sites. In addition, a confidential STI/HIV clinic for CSWs and their stable partners offered group health education, diagnosis and treatment for STIs, HIV counselling and testing, and free lubricating gel and male and female condoms. Women were invited to return in one week for their test results and as needed afterwards. | Serial cross-sectional study design  Rigour score = 2 | Condom use |
| Hamlyn, 2007 [36] | United Kingdom  Adults living with HIV  HIV clinic | L20  HIV services adding SRH services | In July 2004, an audit showed the need for a specific sexual health clinic for HIV-positive patients. Nurse-led STI clinics were set up on both a booked appointment and an emergency walk-in basis. | Serial cross-sectional study design  Rigour score = 2 | Quality of services |
| Jones, 2004 [37] | Zambia  HIV-positive and HIV-negative, sexually active women recruited from VCT sites  Study clinics | L1, L5, L16, L20  HIV services adding SRH services | All women were tested for HIV and screened for STIs. There were three arms: group, individual, and usual care. The first two got the same content, just varied by group or individual setting. The intervention was three-monthly two-hour sessions covering HIV/STIs and risk-reduction strategies, with a focus on group cohesion and skill building. Videos were shown. Participants were given free male and female condoms and vaginal chemical products. The usual care arm received VCT and male and female condoms. | Randomized, controlled trial – individual  Rigour score = 7 | None |
| Jones, 2006 [38] | Zambia  Sexually active, HIV-positive women 18 years and older  Study clinic | L1, L5, L16, L20  HIV services adding SRH services | HIV-positive women were randomized to a group or individual intervention. Participants in the group arm received three sessions about HIV and SRH and were provided with male and female condoms, vaginal lubricants, gels and suppositories. Participants in the individual arm were provided with basic HIV/SRH information, male and female condoms and vaginal lubricants. | Randomized, controlled trial – individual  Rigour score = 8 | Condom use  Contraceptive use |
| Khoshnood, 2006 [39] | China  Pregnant women  Antenatal clinics at two large public hospitals | L7  SRH services adding HIV services | Pregnant women received VCT either individually (control) or as couples (intervention). Pre-test counselling lasted about 15 minutes and covered core topics, including modes of transmission, the window period, prevention, and implications of a positive or negative test result. In the intervention group, women and their male partners received VCT together. In the control group, women received VCT individually and male partners did not receive VCT. Participants returned one week later for their test results and 5-10 minute post-test counselling. | Non-randomized trial – group  Rigour score = 5 | None |
| Kiarie, 2006 [40] | Kenya  Pregnant women  Antenatal clinic | L7  SRH services adding HIV services | At a public ANC clinic, women attending a first ANC visit were given health education in groups of 5-10 and invited to participate. Women returned at their convenience, with or without their partner, for counselling and HIV testing. Women returned after two weeks for follow-up counselling and nevirapine when appropriate. The article focuses on reports of domestic violence after testing. | Pre-post study design  Rigour score = 3 | None |
| King, 1995 [41] | Rwanda  Women attending paediatric and prenatal clinics  Project clinic | L2, L3, L5  HIV services adding SRH services | Women who had received VCT were shown a 15-minute educational video on contraceptive methods, followed by a group discussion to ensure understanding of the information presented. Oral contraceptive pills, injectable progestins, and Norplant were then provided, free of charge, to women who chose to enroll in the family planning programme. | Pre-post study design  Rigour score = 3 | Contraceptive use |
| Kissinger, 1995 [42] | USA  Women living with HIV  HIV outpatient clinic | L9, L24  HIV services adding SRH services | A maternal-child programme was started within an HIV outpatient programme and comprehensive primary care centre. To improve clinic attendance among women, the following interventions were implemented: (1) a separate area in the clinic where the waiting rooms and examination rooms were private and oriented to mothers and children; (2) an increase in the number of female health providers; (3) on-site child care services free of charge; (4) coordination of transportation services; (5) combined paediatric and maternal clinics, merging scheduled visits for mothers and children; (6) daily availability of health care providers for urgent visits; and (7) on-site colposcopy and gynecologic services within the primary care clinic. | Non-randomized trial – individual  Rigour score = 6 | None |
| McCarthy, 1992 [43] | United Kingdom  Women at risk of STIs, including HIV  Women-only study clinic in hospital | L17, L19, L22, L24  Simultaneous | A multidisciplinary, women-only clinic was opened providing HIV testing, HIV follow up, genitourinary screening, colposcopy, and a substance misuse service. Specialist referrals and injection drug user services were available. The clinic was held once a week for two hours, with open access for urgent problems. Staff were all female. | Cross-sectional study design with two comparison groups  Rigour score = 1 | Uptake of HIV testing |
| Peck, 2003 [44] | Haiti  General population  VCT centre | L2, L3, L4, L7, L9, L11, L12, L13, L14, L17, L18, L19  HIV services adding SRH services | Progressive integration of primary care services into VCT. GHESKIO HIV counselling and testing centre opened in 1985; this centre also provided HIV care through on-site adult and paediatric clinics. In 1989, TB services were added. In 1991, STI management was added. In 1993, family planning services and nutritional support for families affected by HIV were added. In 1999, prenatal services for HIV-positive pregnant women (including PMTCT), post-rape services (including counselling, emergency contraception, and post-exposure prophylaxis, including for health care workers accidentally exposed to HIV) were all added. | Serial cross-sectional study design  Rigour score = 1 | Uptake of HIV testing |
| Rasch, 2006 [45] | United Republic of Tanzania  Women presenting after an illegal abortion  Municipal hospital | L1, L2  SRH services adding HIV services | Women with incomplete abortion presenting at a municipal hospital were approached and interviewed using an empathetic approach. Women who revealed having had an illegally induced abortion were characterized as having an unsafe abortion. Women were offered HIV testing, as well as contraceptive counselling and services and counselling about STIs/HIV. Re-counselling and contraceptive services were provided at follow up. Promotion of condoms and double protection was included. | Cross-sectional study design  Rigour score = 2 | Contraceptive use |
| Richardson, 2004 [46] | USA  HIV-positive, sexually active adults  HIV clinics | L19  HIV services adding SRH services | HIV-positive patients at six HIV clinics received safer sex messages in either a gain-framed or loss-framed approach, or they received attention-matched control sessions on adherence to ART. Counselling was brief (3-5 min) and given at all visits, except those dealing with acute illness. The importance of a patient-provider team approach to help patients stay healthy was emphasized. Similar information was included in a brochure. | Randomized controlled trial – group  Rigour score = 5 | Quality of services |
| Semrau, 2005 [47] | Zambia  Pregnant women  Antenatal clinics | L7  SRH services adding HIV services | Women attended a group education session about HIV and PMTCT, and were then offered HIV testing and provided with results. Nevirapine was offered to HIV-positive women at post-test counselling. Participation of male partners was encouraged through community outreach. Couple counselling was particularly encouraged, and many outreach activities specifically targeted men. | Cross-sectional study design  Rigour score = 1 | None |
| Sherr, 2007 [48] | Zimbabwe  General population  Mobile clinics/community sample | L16, L17  Simultaneous | Free HIV counselling and testing and free treatment for other STIs were made available in the study areas through a mobile VCT clinic. Nurse counsellors provided counselling using a systematic approach that emphasized the background of the client and tailored pre- and post-test counselling accordingly. | Prospective cohort  Rigour score = 4 | HIV incidence  Condom use  Uptake of HIV testing |
| Simpson, 1998 [49] | United Kingdom  Pregnant women  Antenatal clinic at main maternity hospital | L7  SRH services adding HIV services | Women attending their first ANC visit were randomly assigned to one of four intervention groups or the control group. Women in the intervention groups were directly offered HIV testing by a midwife (universal policy). Women in the intervention groups got a leaflet, either HIV specific or for all blood tests, and had either minimal or comprehensive discussion of HIV testing with a midwife. Women in the control group were not routinely offered a test and were not given any information about the test unless they asked (standard of care). | Cross-sectional study design  Rigour score = 3 | Uptake of HIV testing  Quality of services |
| Sirivongrangson, 2006 [50] | Thailand  Women living with HIV  Public infectious disease and STI clinics | L19  HIV services adding SRH services | HIV-positive women at an infectious disease clinic and an STI clinic were offered STI screening. Women consenting to STI services underwent a gynecologic examination, were screened for genital ulcers by visual examination, and samples were obtained for STI testing. STI treatment and condoms were provided free of charge. | Cross-sectional study design  Rigour score = 1 | None |
| Stringer, 2007 [51] | Zambia  HIV-positive women who had delivered recently at government primary care clinics and had been tested via PMTCT  Setting not reported | L5  HIV services adding SRH services | Women were randomly assigned to receive either the intra-uterine device or hormonal contraception. All participants underwent pelvic examination. Women who were allocated to the hormonal contraception arm were allowed to choose between oral contraceptive pills or depo injections, both provided at three-month intervals. Condoms were offered at each follow-up visit. | Randomized, controlled trial – individual  Rigour score = 5 | None |
| Stringer, 2001 [52] | USA  Pregnant women  Antenatal clinics | L7  SRH services adding HIV services | At eight ANC clinics, a new policy made HIV testing part of routine ANC for all women. Previously, at their initial prenatal encounter, all women had been given written information about HIV testing and were counselled by the maternity providers. At that time, they were asked to decide whether they wanted HIV testing or not. Under the new policy, maternity providers referred women to written materials (a one-page guide about HIV and pregnancy) about HIV and notified them that an HIV test would be performed as part of the routine battery of prenatal lab tests. At this time, all women signed consent for treatment, and those who agreed to HIV testing signed a consent form specifically for the HIV test. | Serial cross-sectional design  Rigour score = 3 | Uptake of HIV testing |
| Stringer, 2003 [53] | Zambia  Pregnant women  District health facilities providing comprehensive obstetric services (ANC, delivery and postnatal care) | L7  SRH services adding HIV services | All women received PMTCT and nevirapine educational messages in a group session. Two strategies for nevirapine administration were then tested: (1) a targeted strategy with women counselled and offered voluntary HIV testing; those who accepted testing and were HIV positive were offered nevirapine; and (2) a universal strategy with women counselled but not offered HIV testing; all women were offered nevirapine. | Randomized controlled trial – group (with crossover)  Rigour score = 4 | None |
| van't Hoog, 2005 [54] | Kenya  Pregnant women  Government hospital | L7  SRH services adding HIV services | Client flow was revised to integrate counselling, HIV testing, and dispensing of single-dose nevirapine into routine antenatal services. The number of facilities providing PMTCT services was expanded to increase district-wide coverage. Facility staff were trained in PMTCT. Women received a strengthened package of PMTCT services, including ferrous sulfate, folic acid, presumptive malaria treatment, routine syphilis testing, opt-in VCT, and general info about PMTCT. Women who tested HIV positive were asked to return for another counsellor visit and were given maternal and infant nevirapine. | Serial cross-sectional study design  Rigour score = 1 | Uptake of HIV testing |
| Wingood, 2004 [55] | USA  Women living with HIV  Setting NR; Women recruited from HIV clinics | L20  HIV services adding SRH services | Four-weekly four-hour interactive group sessions emphasizing gender, maintaining supportive networks, HIV risk behaviours and myths, communication and condom use skills, and healthy relationships. Each session of 8-10 participants was facilitated by a female health educator and a female HIV-positive peer educator. A comparison group received four four-hour group sessions on medication adherence, nutrition, and provider interaction skills. | Randomized controlled trial – individual  Rigour score = 8 | STI incidence  Condom use |
| Xu, 2002 [56] | Thailand  HIV-negative women attending family planning and post-partum clinics  Study clinic | L2, L7  HIV services adding SRH services | Trained nurse counsellors provided pre- and post-test HIV counselling that lasted 20 to 45 minutes each. Condom use was recommended if husband’s HIV status was positive or unknown. Women were shown how to use condoms and were given condoms to take home. Partner HIV testing was recommended and the cost reimbursed if the husband tested. | Time series study design  Rigour score = 3 | Condom use  Uptake of HIV testing |

*Types of linkages refer to cells in Table 1 and indicate which types of HIV interventions and which types of SRH interventions were included in the study intervention. Cells in Table 1are labeled L1 through L25 (for linkage type 1 through 25), starting at the upper left hand corner and moving across, then down. L1 is therefore family planning linked with HIV prevention, education and condoms; L2 is family planning linked with HIV testing; etc.
